# Atovaquone and Berberine Chloride Reduce SARS-CoV-2 Replication In Vitro

**DOI:** 10.3390/v13122437

**Published:** 2021-12-04

**Authors:** Bruno A. Rodriguez-Rodriguez, Maria G. Noval, Maria E. Kaczmarek, Kyung Ku Jang, Sara A. Thannickal, Angelica Cifuentes Kottkamp, Rebecca S. Brown, Margaret Kielian, Ken Cadwell, Kenneth A. Stapleford

**Affiliations:** 1 Department of Microbiology, New York University Grossman School of Medicine, New York, NY 10016, USA; Bruno.Rodriguez-Rodriguez@nyulangone.org (B.A.R.-R.); Maria.Noval@nyulangone.org (M.G.N.); kaczmarek@ecohealthalliance.org (M.E.K.); Kyungku.Jang@nyulangone.org (K.K.J.); Sara.Thannickal@nyulangone.org (S.A.T.); Ken.Cadwell@nyulangone.org (K.C.); 2 Kimmel Center for Biology and Medicine at the Skirball Institute, New York University Grossman School of Medicine, New York, NY 10016, USA; 3 Department of Medicine, New York University Grossman School of Medicine, New York, NY 10016, USA; Angelica.Kottkamp@nyulangone.org; 4 Department of Cell Biology, Albert Einstein College of Medicine, Bronx, NY 10461, USA; rebeccashbrown@gmail.com (R.S.B.); margaret.kielian@einsteinmed.edu (M.K.)

**Keywords:** antiviral, coronavirus, atovaquone, berberine chloride

## Abstract

Epidemic RNA viruses seem to arise year after year leading to countless infections and devastating disease. SARS-CoV-2 is the most recent of these viruses, but there will undoubtedly be more to come. While effective SARS-CoV-2 vaccines are being deployed, one approach that is still missing is effective antivirals that can be used at the onset of infections and therefore prevent pandemics. Here, we screened FDA-approved compounds against SARS-CoV-2. We found that atovaquone, a pyrimidine biosynthesis inhibitor, is able to reduce SARS-CoV-2 infection in human lung cells. In addition, we found that berberine chloride, a plant-based compound used in holistic medicine, was able to inhibit SARS-CoV-2 infection in cells through direct interaction with the virion. Taken together, these studies highlight potential avenues of antiviral development to block emerging viruses. Such proactive approaches, conducted well before the next pandemic, will be essential to have drugs ready for when the next emerging virus hits.

## 1. Introduction

RNA viruses include significant human pathogens with pandemic potential. Among these viruses are influenza viruses, coronaviruses, flaviviruses, and alphaviruses. The current outbreak of SARS-CoV-2 is a prime example of how these RNA viruses, if given the right conditions, can spread rapidly throughout the world. Although effective vaccines for SARS-CoV-2 were eventually developed, the availability of potent and orally available antivirals at the beginning would have had an immense impact on the trajectory of the outbreak, potentially saving thousands of lives and diminishing the socioeconomic burden. However, over one year later, there are no approved oral antivirals for SARS-CoV-2 or for many RNA viruses. This problem and the fact that RNA viruses continue to emerge highlight the need to develop broad-spectrum antivirals to RNA viruses before the next pandemics strike.

The absence of a SARS-CoV-2 antiviral compound is not for a lack of trying. At the onset of the pandemic, many groups tested countless compounds in the hope of developing SARS-CoV-2 antivirals [1,2,3,4,5]. These studies identified potential candidates, several of which are or were in clinical trials. These compounds include Remdesivir [6], a known coronavirus antiviral that is used in the clinical setting, as well as novel 3CL protease inhibitors [7]. However, while these compounds are potent antivirals they must be administered intravenously and are therefore not effective for large scale distribution or as a prophylaxis during a pandemic. Moreover, in preparation for the next pandemic, we should attempt to develop compounds that are broad-spectrum antivirals, potentially inhibiting multiple RNA viruses [4]. This idea will not only allow for the development of potent antivirals but suggests these drugs may be targeting conserved, essential steps in the viral life cycle that are shared by multiple viruses. Therefore, we can use these compounds to understand the fundamental mechanisms of RNA virus biology.

In this study, we screened a panel of FDA-approved compounds that have been shown to possess antiviral or antimicrobial activity for their ability to inhibit SARS-CoV-2. We found that while most compounds were ineffective against SARS-CoV-2, atovaquone, a pyrimidine biosynthesis inhibitor and antiparasitic drug was able to reduce SARS-CoV-2 replication in Vero E6 and human lung cells. In addition, we found that berberine chloride, a natural, plant-based compound was able to reduce SARS-CoV-2 infectivity and block SARS-CoV-2 replication in vitro. Together, these studies identify several well-tolerated compounds that have the potential to be used as broad-spectrum antivirals in the case of the next pandemic.

## 2. Materials and Methods

### 2.1. Cells and Viruses

Vero E6 (ATCC CRL-1586) and Calu-3 (ATCC HTB-55) cells were maintained in Dulbecco’s modified eagle media (DMEM, Corning) supplemented with 10% fetal bovine serum (FBS, Atlanta Biologicals (Flowery Branch, GA USA)), 10 mM HEPES pH 7.4 (Gibco, Gaithersburg, MD, USA), and 1% nonessential amino acids (NEAA, Corning, Corning, NY, USA) at 37 °C with 5% CO_2_. All cells were verified mycoplasma free.

SARS-CoV-2, isolate USA-WA1/2020 [8] (BEI resources #NR52281, a gift from Mark Mulligan at the NYU Langone Vaccine Center) and icSARS-CoV-2-mNG (isolate USA/WA/1/2020, obtained from the World Reference Center for Emerging Viruses and Arboviruses at UTMB [9]) were amplified once in Vero E6 cells (P1 from the original stock). Briefly, a 90–95% confluent T175 flask of Vero E6 (1 × 10^7^ cells) was infected with 10 μL of the BEI stock in 3 mL or 50 μL of icSARS-CoV-2-mNG in 5 mL of infection media (DMEM, 2% FBS, 1% NEAA, and 10 mM HEPES) for 1 h. After 1 h, 15 mL or 20 mL of infection media was added to the inoculum and cells were incubated 72 h at 37 °C and 5% CO_2_. After 72 h, the supernatant was collected, and the monolayer was frozen and thawed once. The supernatant and cellular fractions were combined, centrifuged for 5 min at 1200 rpm, and filtered using a 0.22 μm Steriflip (Millipore, Burlington, MA, USA). Viral titers were determined by plaque assay in Vero E6 cells as described below. Chikungunya virus (CHIKV) was rescued from the CHIKV strain 06-049 (AM258994) infectious clone as previously described [10]. All work with SARS-CoV-2 and CHIKV was completed under BSL3 biosafety conditions at the NYU Grossman School of Medicine.

### 2.2. Plaque Assays

Virus-containing supernatants were subject to six 10-fold serial dilutions in DMEM. A total of 100 μL of each dilution was added to a monolayer of Vero E6 cells (~200,000 cells per well in a 24-well plate) for 1 h at 37 °C. After incubation, an agarose overlay of 0.8% agarose in DMEM with 2% FBS was added, and cells incubated for 72 h at 37 °C and 5% CO_2_. Each well was then fixed with 10% formalin for 1 h, the agarose plug removed, and plaque visualized with crystal violet (10% crystal violet, 20% ethanol). Plaques were counted on the two lowest countable dilutions to determine viral titers and expressed as plaque forming units per milliliter (PFU/mL).

### 2.3. Compound Treatment

Vero E6 (30,000 cells/well) were plated 24 h prior to compound treatment in 96 well plates. For Calu-3 cells, 30,000 cells were plated, and cells were monitored for 5–9 days until they reach confluency. Media was changed every 3 days for Calu-3 cells. The day of infection, the compounds (Table 1) and carriers were subject to 2-fold serial dilutions in infection media (DMEM, 2% FBS, 1% NEAA, 10 mM HEPES) and cells were pretreated with compound and carrier dilutions for 2 h. Cells were then infected with either SARS-CoV-2 (Vero E6) or icSARS-CoV-2-mNG (Calu-3) at an MOI of 0.1. Virus was diluted in infection media containing drug at the same concentration as the pretreatment. Cells were incubated with virus for 1 h at 37 °C and 5% CO_2_. After 1 h, cells were washed twice with phosphate-buffered saline (PBS) and media containing compound or carrier was added to each well. Cells were incubated for 36 h. Virus titers from supernatants were quantified using a plaque assay as described above.

### 2.4. Cell Cytotoxicity Assays

A total of 30,000 cells/well were seeded into a 96-well plate in DMEM supplemented with 2% FBS and 1% NEAA. Further, 2-fold serial dilutions of each compound or the carrier control were made in DMEM containing 2% FBS. Cells were incubated with either drug or carrier control for 36 h. Cell viability was measured by MTT asssay using the CellTiter96 Non-Radioactive Cell Proliferation Assay (Promega, Madison, WI, USA) or lactate dehydrogenase (LDH) release assay using the CytoTox96 Non-Radioactive Cytotoxicity Assay (Promega, Madison, WI, USA) following the manufacturer’s instructions.

### 2.5. Cell Proliferation Experiment

A total of 30,000 cells/well were seeded into a 96-well flat transparent black plate in DMEM supplemented with 2% FBS and 1% NEAA. In addition, 2-fold serial dilutions of each compound or the carrier control were made in DMEM containing 2% FBS. Cells were incubated with either drug or carrier control for 36 h. After 36 h cells were treated as follows: (i) carrier control condition, media was removed and replenished with fresh media (DMEM containing 2% FBS); (ii) mock condition, media with drug was removed and washed once with PBS and replenished with fresh media or (iii) rescue treatment, media with drug was removed, washed once with PBS and replenished with media containing 100 μM uridine (Sigma, St. Louis, MO, USA). Each treatment was incubated for 24 h and then fixed for 1 h with 4% paraformaldehyde. After fixation, cells were permeabilized with 0.25% Triton X-100, stained with 4′,6-Diamidino-2-Phenylindole, Dihydrochloride (DAPI, Invitrogen, Waltham, MA, USA), and quantified on a Cell-Insight CX7 High-content microscope (Thermo-Scientific, Waltham, MA, USA) using the HCS Navigator Version 6.6.1 (Thermo-Scientific, Walthan, MA, USA).

### 2.6. Human Airway Epithelial (HAE) Cultures and Infections

EpiAirway tissues were obtained from MatTek Life Sciences (Part No. AIR-100; Sterile/Lot #33106). The 3D airway models were equilibrated overnight in assay medium AIR-100-ASY (MatTek Life Sciences, Ashland, MA, USA). After equilibration, models were pre-treated with atovaquone or DMSO by placing media containing compound or carrier on the basolateral side of each model and incubating for 2 h at 37 °C and 5% CO_2_. Prior to viral infection, mucus was washed from the apical surface of the airway model by adding 400 mL TEER buffer (PBS with calcium and magnesium) to the apical surface of each EpiAirway tissue and incubating for 30 min. TEER buffer was then removed, and this process repeated once more. Models were then infected apically with icSARS-CoV-2-mNG at an MOI of 0.5 and incubated for 2 h at 37 °C and 5% CO_2_. After 2 h, the viral inoculum was removed, and the apical surface washed three times as described above. Every 24 h media containing atovaquone or DMSO was replaced on the basolateral side of each model. Timepoints were collected every 24 h for 72 h by adding 100 μL TEER buffer to the apical side of the model and incubating for 30 min before collecting the wash. Virus titers from washes were quantified by plaque assay.

### 2.7. Human Colonic Organoid Cultures and Infections

Human colonic organoids were cultured as described previously [26,27]. Non-inflamed pinch biopsies of ascending colon were obtained with consent from an ulcerative colitis patient (C1, 33-year-old male) or an adult healthy subject (C2, 34-year-old male) undergoing surveillance colonoscopy, using 2.8-mm standard biopsy forceps, after protocol review and approval by the NYU Grossman School of Medicine Institutional Review Board (Mucosal Immune Profiling in Patients with Inflammatory Bowel Disease; S12-01137). The inflammation status of tissue was confirmed by pathological examination. All pinch biopsies were collected in ice-cold complete RPMI (RPMI 1640 medium supplemented with 10% FBS, penicillin/streptomycin/glutamine, and 50 μM 2-mercaptoethanol). They were incubated in Gentle Cell Dissociation Reagent (Stemcell Technologies, Vancouver, Canada) on ice for 30 min, followed by vigorous pipetting to isolate crypts. The crypts were embedded in 30 μL of Matrigel and cultured with Human IntestiCult™ Organoid Growth Medium (OGM) (Basal Medium and Organoid Supplement, Stemcell Technologies, Vancouver, Canada) supplemented with 100 IU Penicillin and 100 μg/mL Streptomycin (Corning, Corning, NY, USA) and 125 μg/mL Gentamicin (Thermo Fisher, Waltham, MA, USA), herein refereed as expansion medium. The culture medium was changed every 2–3 days. For passing human organoids, 10 μM Y-27632 were added for the first 2 days. To generate 2D colonic monolayers, mature human colonic organoids grown with the expansion media were digested into single cells using TrypLE Express (Thermo Fisher, Waltham, MA, USA) and seeded into Matrigel-coated 96-well culture plate (Corning) in Y-27632-supplemented expansion medium at 150,000 cells/well for the first 2 days. For differentiation, the 2D monolayers were cultured with 1:1 mix of Human IntestiCult™ OGM Basal Medium and DMEM/F-12 (Thermo Fisher, Waltham, MA, USA) in the presence of 100 IU Penicillin and 100 μg/mL streptomycin, 125 μg/mL gentamicin and 2 mM L-glutamine (Corning, Corning, NY, USA), herein referred as differentiation medium. The differentiation media were changed every day. The 2D monolayers grown with the differentiation media for 7 days were infected with SARS-CoV-2-mNG at a MOI of 4 for 1 h at 37 °C. Following incubation, the 2D monolayer was washed four times with phosphate buffered saline and differentiation media was added for the indicated time. Viral titers in the monolayer supernatants were quantified by plaque assay.

### 2.8. Berberine Chloride (BBC) Cell and Direct Virus Treatment Assay

BBC was dissolved as per previously published [28]. For BBC cell treatment, Vero E6 cells (30,000 cells/well) were seeded the day before in a 96-well plate. The day of infection, cells were infected with SARS-CoV-2 (MOI = 0.1) or CHIKV (MOI = 1) for 1.5 h, washed three times with PBS, and complete media was added back to the cells. At 6 hpi, cells were washed three times with PBS and media containing DMSO or BBC was added to the cells. Supernatants were collected 24 h later and viral titers were quantified by plaque assay. Calu3 cells were seeded and infected the same as Vero E6 cells, yet supernatants were harvested at 36 hpi.

For direct virus treatment, SARS-CoV-2 (MOI = 0.1) and CHIKV (MOI = 1) were incubated directly with DMSO or BBC for 1 h at 37 °C. Following incubation, virus was then used directly for plaque assay and incubated on Vero E6 cells. After 1 h incubation, the cells were washed three times with PBS to remove any BBC that may interfere with the plaque assay. An agarose overlay was then added, and the plaque assay developed 72 h later as described above.

### 2.9. Data Analysis and Statistics

All statistical analysis and data visualization was performed using GraphPad Prism (Version 9.1.2) All experiments were completed at least two independent times with internal technical duplicates. The specific statistical test used, and the N can be found in each figure. *p* > 0.05 is considered non-significant.

## 3. Results

### 3.1. Screen of FDA-Approved Compounds against SARS-CoV-2

**Figure 1:**
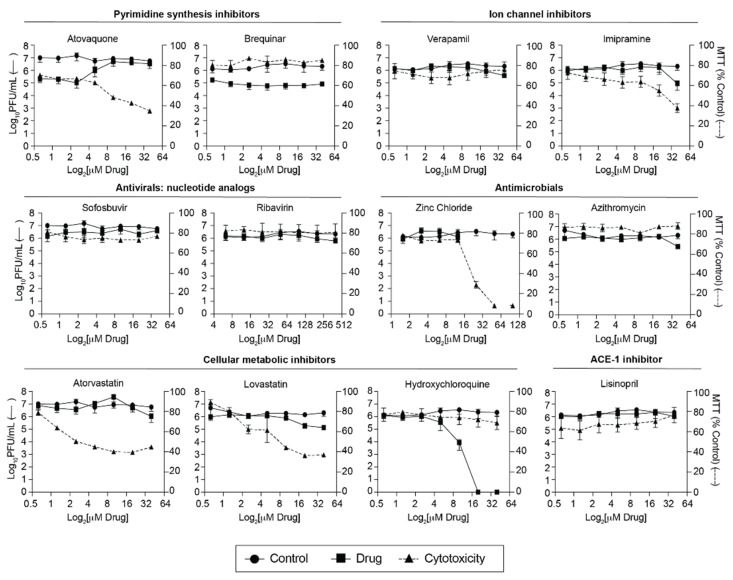
Screen of FDA-approved compounds against SARS-CoV-2 on Vero E6 cells. Vero E6 cells were pretreated for 2 h with increasing concentrations of the indicated drug or vehicle control and then infected with SARS-CoV-2 at an MOI of 0.1 At 36 h post infection (hpi), supernatants were collected, and infectious particles were quantified by plaque assay on Vero E6 cells. For MTT assays, Vero E6 cells were treated with compound or vehicle for 36 h and harvested for analysis. Data represents the mean and range of two independent experiments performed in technical duplicates.

As a screen for anti-SARS-CoV-2 compounds, we tested 12 compounds (Table 1) in Vero E6 cells over multiple concentrations. We specifically chose compounds with known antiviral or antimicrobial activity, hypothesizing that these may also act against SARS-CoV-2. These concentrations spanned a range of known effective concentrations from previous studies (Table 1). To capture the effect of the compound at all stages of viral replication, cells were pretreated for two hours with compound or carrier, during and after viral infection. Since the drugs we chose target a wide range of pathways we used a highly susceptible cell line, Vero E6, to capture all possible antiviral effects. Of the 12 compounds we tested, hydroxychloroquine and azithromycin, have been implemented as treatments early during the COVID-19 pandemic. In our screen, hydroxychloroquine substantially decreased SARS-CoV-2 titers while having low levels of toxicity (Figure 1). On the other hand, azithromycin, which was frequently prescribed alongside hydroxychloroquine to treat COVID-19, had no significant effect on viral titers in Vero E6 cells. Hydroxychloroquine and azithromycin have now been shown to have negative side effects and low to no efficacy in COVID-19 patients [29,30]. These results highlight that numerous, potentially efficacious, drugs should be tested in clinical settings because in vivo outcomes are not always reflected in in vitro systems and vice versa. Of the other compounds tested, many had no impact on SARS-CoV-2 infection (sofosbuvir, ribavirin, lisinopril, and verapamil) or tended to be cytotoxic and not a good candidate for an antiviral in vitro (imipramine, atorvastatin, lovastatin, and zinc chloride).

**Figure 2:**
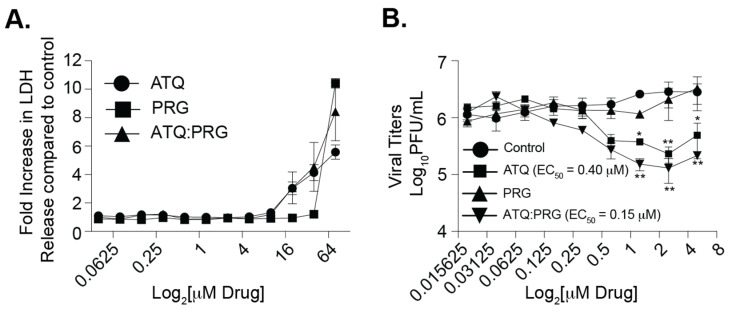
Effect of Malarone on SARS-CoV-2 replication. (**A**) Cell cytotoxicity after 36 h compound treatment quantified by LDH release assay. Two independent experiments with internal technical duplicates. (**B**) Vero E6 cells were pretreated with increasing concentrations of atovaquone (ATQ), proguanil (PRG), or the combination of ATQ and PRG (ATQ:PRG), infected with SARS-CoV-2 at a MOI of 0.1. Viral titers in the supernatant were quantified at 36 hpi by plaque assay. Data represent the mean and range of two independent experiments with internal technical duplicates. * *p* < 0.05, ** *p* < 0.01. Two-way ANOVA.

However, we found that atovaquone and brequinar reduced SARS-CoV-2 titers compared to the vehicle control, while maintaining cell viability (Figure 1). These compounds are pyrimidine synthesis inhibitors and have been shown to possess antiviral activity against RNA viruses [13,16,17]. In particular, we have shown that atovaquone can inhibit Zika and chikungunya virus replication in vitro [13] and therefore, we decided to continue with this compound for our study. Interestingly, similar to what we have seen previously for Zika virus, we found that atovaquone’s anti-SARS-CoV-2 effect is relieved at the three highest concentrations tested. At these concentrations, atovaquone is more toxic as measured by a mitochondrial assay and crystals formed in the media. This reduction in MTT assay readout may be explained by the fact that atovaquone is a direct mitochondria inhibitor, as we do not see this level of toxicity when we measure cell viability using a lactate dehydrogenase release assay (Figure 2A). However, crystal aggregation can result in a lower effective concentration because solute is removed out of the media solution. The lower effective concentration may explain why viral outgrowth is detected at high atovaquone concentrations. Nevertheless, our initial screen identifies atovaquone as antiviral compounds against SARS-CoV-2 in Vero E6 cells.

### 3.2. Atovaquone and Proguanil Hydrochloride Function Together to Reduce SARS-CoV-2 Infectious Particle Production

Atovaquone is a well-tolerated antiparasitic drug used in the treatment of parasitic infections [20,31,32]. As a malaria prophylaxis, atovaquone is given in combination with proguanil-chloride (Malarone^®^) which are thought to act synergistically against the malaria parasite [33]. Therefore, we asked whether this combination of drugs which is well-tolerated in humans could affect SARS-CoV-2 replication in vitro (Figure 2). We pretreated Vero E6 cells with each compound individually as well as together, infected cells with SARS-CoV-2, and then added compound back during the entire infection. We found that as before, atovaquone inhibited SARS-CoV-2 replication in a dose-dependent manner while proguanil-chloride had no antiviral effect (Figure 2B). Interestingly, when we combined the two compounds, we found a slight further reduction in viral titers suggesting that atovaquone is still functional in the presence of proguanil-chloride and proguanil-chloride may enhance atovaquone function. Importantly, the reduction in viral titers was not due to cell toxicity of the compounds as the concentrations used were well within the range of cell cytotoxicity as measured by LDH release (Figure 2A).

### 3.3. Atovaquone Affects Cell Proliferation

**Figure 3:**
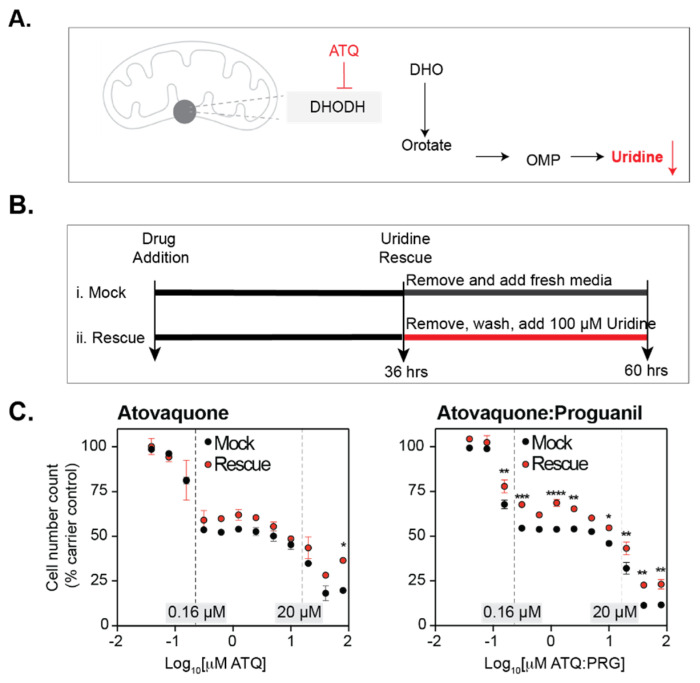
Effect of atovaquone and atovaquone-proguanil on cell proliferation. (**A**) Diagram of atovaquone mode of action. (**B**) Schematic of the experimental setup. Briefly, Vero E6 cells were grown in the presence of each drug or carrier for 36 h. Next, cells were treated as follows: (i) mock treatment, media with drug was removed, the monolayer was washed once with PBS and fresh media added; (ii) rescue treatment, media with drug was removed, the monolayer was washed once with PBS, and then media with 100 μM uridine was added. For both conditions, cells were incubated for 24 h. (**C**) Effect of atovaquone and malarone on cellular proliferation. Each panel indicates the total cell count expressed as % of carrier control at different drug concentrations. Dashed lines indicate a range of concentrations from 0.16 to 20 μM where the dose response effect was stable. *p* values were calculated using a two-Way ANOVA with Bonferroni correction. (* *p* < 0.05, ** *p* < 0.01, *** *p* < 0.001, **** *p* < 0.0001).

The initial drug screen revealed that atovaquone is a potential candidate for a SARS-CoV-2 antiviral drug. Atovaquone targets pathways involved in the mitochondrial electron transport chain, and inhibits dihydroorotate dehydrogenase (DHODH), a key enzyme in the pyrimidine biosynthesis pathway [34,35,36] (Figure 3A). The depletion of nucleotides is associated with the cell arrest response in cells [37,38]. Since atovaquone inhibits DHODH, an important enzyme in the pyrimidine biosynthesis pathway, we hypothesized that atovaquone’s effect on SARS-CoV-2 may be related to its effect on cellular metabolism. This phenotype is known to be reversible [37,38]. Therefore, to test whether the antiviral effect we detected was related to cell arrest, we treated cells with atovaquone or atovaquone:proguanil at a range of concentrations and rescued them by adding back uridine (Figure 3B,C). Across this range we found three stages. At the lowest concentrations we saw cell loss (0.16 μM and below) and no effect of uridine rescue on this loss (Figure 3C). However, in this same range uridine does significantly rescue cell proliferation in atovaquone:proguanil. The second stage is characterized by cell arrest (0.16 μM–20 μM), where cell number does not change and uridine does rescue overall cell number to levels seen at 0.16 μM of drug, but not higher. Finally, the highest concentrations of atovaquone and atovaquone:proguanil resulted in cell death, but uridine did rescue this effect to some degree. Our entire antiviral dose-response is encompassed in the 0.16 μM–20 μM range where cell arrest occurs. This suggests that while cell arrest may have an effect on viral replication, there is an additional and separate effect of atovaquone on SARS-CoV-2.

### 3.4. Atovaquone Reduces SARS-CoV-2 Infection in Calu-3 Cells But Not in Human Airway Epithelial Cultures or Colonic Organoids

**Figure 4:**
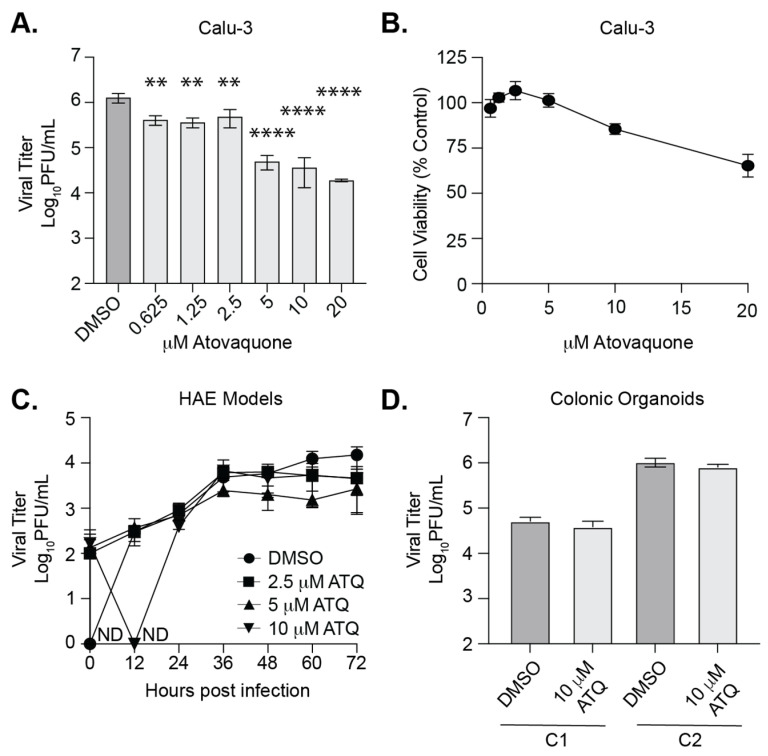
Effect of atovaquone in Calu-3 cells and human airway epithelial cultures and human colonic organoids. (**A**) Calu-3 cells were pretreated with increasing concentrations of ATQ for 2 h, infected with SARS-CoV-2-mNG at an MOI of 0.1, and the compound added back. Viral titers were quantified in the supernatant at 36 hpi by plaque assay. Data represent the mean and range of two independent experiments with internal duplicates. Two-way ANOVA. ** *p* < 0.01, **** *p* < 0.001. (**B**) Atovaquone cytotoxicity on Calu-3 cells by MTT assay. (**C**) Human airway epithelial cultures were pretreated with ATQ for 2 h, infected with icSARS-CoV-2-mNG, and the compound replaced. Viral titers were quantified in the culture supernatant by plaque assay. (**D**) Human colonic organoid cultures (C1 and C2) were pretreated with 10 μM ATQ for 2 h, infected with SARS-CoV-2-mNG at MOI of 0.5, and cultured in the presence of compound for 36 h. Viral titers in the supernatant were quantified by plaque assay. ND = not detected (below limit of detection).

Our initial screen was completed in highly susceptible Vero E6 cells. Therefore, we tested the antiviral properties of atovaquone in Calu-3, human lung epithelial cells (Figure 4). Since SARS-CoV-2 is a respiratory virus found in the airway and lungs, this cell line is a more relevant model for infection. We found that atovaquone significantly inhibited SARS-CoV-2 replication in Calu-3 cells (Figure 4A, EC_50_ = 10.21 μM) at concentrations with minimal cytotoxicity (Figure 4B). Interestingly, we did not find the synergistic effects of atovaquone with proguanil in Calu3 as we performed in Vero E6 cells (data not shown). Together with our results from Figure 2, these data show that atovaquone is able to reduce SARS-CoV-2 infectious particle production in multiple mammalian cell lines, including human lung cells.

However, while cell lines are useful models and tractable for manipulation, they lack the multi-faceted properties of an organ, e.g., cell-type variability and 3D structure. Further, 3D human airway epithelial (HAE) cultures provide a more realistic infection model, and we therefore employed them in our drug test. We pretreated HAE cultures with DMSO or increasing concentrations of atovaquone for 2 h, infected the HAEs with SARS-CoV-2, and added back media containing atovaquone. We found that SARS-CoV-2 replicated in the DMSO treated cultures with increasing viral titers in the supernatant over the course of three days (Figure 4C). However, we observed that cultures treated with atovaquone, particularly 5 μM of atovaquone, seemed to plateau around 36 h with no dramatic increases in shedding virus. While these data were not statistically significant, we found this data striking and it could suggest that atovaquone is interfering with SARS-CoV-2 replication in HAEs. Finally, given that SARS-CoV-2 can infect the human gut and has been shown to infect human gut organoids [39,40,41], we investigated whether atovaquone could inhibit SARS-CoV-2 replication in human 2D colonic organoids (Figure 4D). We generated two colonic 2D organoid cultures (C1 and C2), treated these cultures with atovaquone and infected each culture with SARS-CoV-2. We collected the supernatant 72 h later and quantified viral titers by plaque assay. We found that atovaquone showed no antiviral activity in human gut organoids, suggesting that the cell type and organ model play important roles in drug function. Taken together, these results show that atovaquone can reduce SARS-CoV-2 replication human lung cells, yet it does not possess potent antiviral activity in human airway epithelial cultures or colonic organoid models.

### 3.5. Berberine Chloride Inhibits SARS-CoV-2 Replication and Infectivity

**Figure 5:**
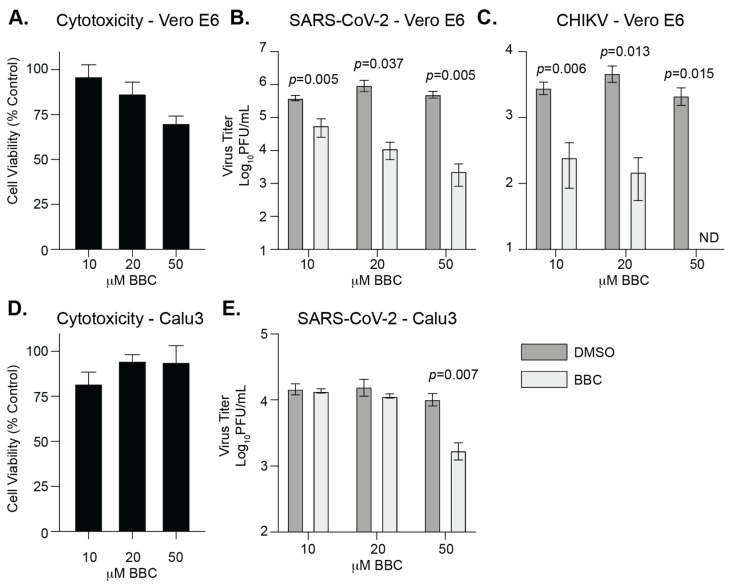
Berberine chloride inhibits SARS-CoV-2 replication in vitro. (**A**) Cytotoxicity of BBC in Vero E6 cells measured by MTT assay. N = 2 with internal triplicates. Vero E6 cells were infected with SARS-CoV-2 (MOI = 0.1) (**B**) or CHIKV (MOI = 1) (**C**) BBC or DMSO was added at 6 hpi and supernatants were harvested at 24 hpi. Viral titers were quantified by plaque assay. Data represent the mean and range of two independent experiments with internal duplicates. Students *t*-test. (**D**) Cytotoxicity of BBC in Calu3 cells measured by MTT assay. N = 2 with internal duplicates. (**E**) Calu3 cells were infected with SARS-CoV-2 (MOI = 0.1) and BBC was added at 6 hpi. Supernatants were harvested at 36 hpi. Viral titers were quantified by plaque assay. Data represent the mean and SEM. N = 3 with internal duplicates. Students *t*-test. ND = not detected (below limit of detection).

In addition to FDA-approved compounds we were also interested in testing compounds used in holistic medicine for anti-SARS-CoV-2 activity. Berberine chloride (BBC) is an orally bioavailable plant-derived molecule that has been shown to have antiviral activity [28,42,43] and can enhance the antibacterial activity of some antibiotics [44,45]. Given the role of BBC in blocking alphavirus infection [28,46], we hypothesized that this molecule may work against SARS-CoV-2 as well. To begin, we infected Vero E6 cells with SARS-CoV-2 and 6 hours post infection added increasing concentrations of BBC or DMSO. We then quantified viral particles in the supernatant at 24 hpi (Figure 5A,B). As a control, we used CHIKV, an alphavirus that has been shown to be sensitive to BBC (Figure 5C) [28,46]. We found that BBC was able to reduce SARS-CoV-2 replication in Vero E6 cells in a dose-dependent manner similar to that of CHIKV. To expand these findings to a human and physiologically relevant cell line, we performed the same infections in Calu3 cells (Figure 5D,E). Interestingly, we found SARS-CoV-2 infection to be inhibited only at the highest concentration tested. These data suggest that in terms of SARS-CoV-2 infection, BBC may have a different effective concentration in Calu3 cells.

**Figure 6:**
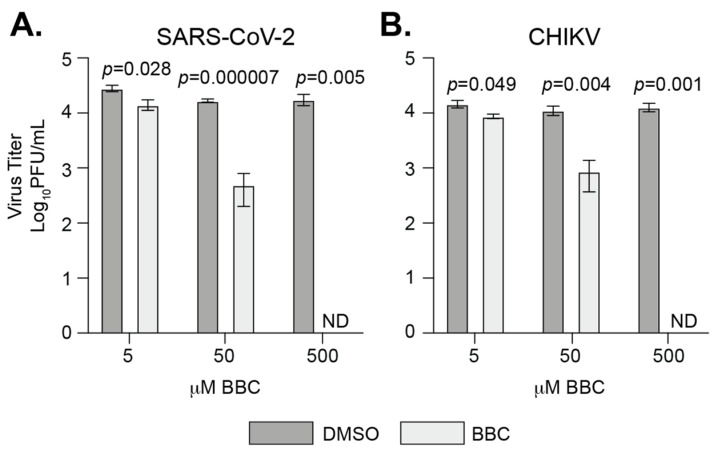
Berberine chloride inhibits SARS-CoV-2 infection. (**A**) SARS-CoV-2 (MOI = 0.1) or (**B**) CHIKV (MOI = 1) were incubated with BBC or DMSO for 1 h at 37 °C. Infectious particles were quantified by plaque assay on Vero E6 cells. Data represent the mean and range of two independent experiments with internal duplicates. Students *t*-test. ND = not detected (below limit of detection).

In addition, it has been shown that BBC can directly reduce the infectivity of free alphavirus particles [28]. Therefore, we tested whether BBC could do the same for SARS-CoV-2 (Figure 6). We mixed SARS-CoV-2 and CHIKV directly with BBC for 1 h at 37 °C and quantified the remaining infectious virus by plaque assay. We found that BBC was able to directly inhibit CHIKV, as published previously [28]. In addition, we found that BBC was able to directly inhibit SARS-CoV-2, suggesting that BBC is interacting with the SARS-CoV-2 particle via an unknown mechanism. Together, these results show that BBC is a broad-spectrum antiviral compound against SARS-CoV-2 and alphaviruses, and functions through multiple modes of action.

## 4. Discussion

The SARS-CoV-2 pandemic will not be the last RNA virus outbreak. Therefore, it is critical that we begin to develop effective antivirals against current and future RNA viruses. In this study, we tested a panel of FDA-approved compounds that have been shown to have antiviral or antimicrobial activities. Similar to what many other researchers have found, we observed that most of these compounds had no effect against SARS-CoV-2 [5]. This observation is interesting, as it suggests that SARS-CoV-2 may not utilize cellular pathways that other RNA viruses exploit, providing a look into the fundamental biology of SARS-CoV-2 infections. On the other hand, the fact that hydroxychloroquine is a potent anti-SARS-CoV-2 molecule in vitro, yet has no role in vivo, raises the question as to whether some of the compounds that have no effect in vitro may indeed be effective in humans. This question suggests that the platform, cell lines, and models used to screen these compounds are important. Indeed, Dittmar et al. [1] found far more active anti-SARS-CoV-2 compounds when they screened them in human Huh7.5 cells as opposed to Vero E6 cells. Given these findings, it will be crucial to also screen potential antiviral compounds in multiple human cell lines and models that are relevant to the clinical presentation of the infection and the pathogenesis of each virus of interest.

Of the compounds tested, we found that both atovaquone and brequinar were able to reduce SARS-CoV-2 replication in Vero E6 cells. Both of these molecules are pyrimidine biosynthesis inhibitors and have been shown to inhibit the replication of SARS-CoV-2 and other viruses [13,16,17,47,48,49,50,51]. These results suggest that pyrimidine biosynthesis is specifically important for SARS-CoV-2 biology since ribavirin, a purine biosynthesis inhibitor, had no impact on SARS-CoV-2 replication. However, one question that remains unanswered is whether pyrimidine biosynthesis inhibitors will work in humans or if these phenotypes are cell-culture specific. Indeed, little or no antiviral activity of pyrimidine biosynthesis has been observed in vivo, a result thought to be mediated by the availability of uridine in the circulation [52]. Nonetheless, there are several clinical trials underway to address the antiviral role of atovaquone in humans and shed light on these questions. In addition, atovaquone affects cell proliferation at a range of concentrations where we see antiviral activity which may also contribute to its antiviral properties. Finally, we found that atovaquone is able to reduce the replication of SARS-CoV-2 in human lung cells and is able to slow infectious virus production in human airway epithelium cultures, yet had no impact in intestinal organoids. These results confirm the anti-SARS-CoV-2 activity of atovaquone as found in several previous studies [47,48]. However, interestingly, in contrast to the study by Carter-Timofte et al., who find a striking reduction in SARS-CoV-2 infection (approximately 6 logs in Vero cells and 4 logs in Calu3 cells), we observed roughly a 2-log decrease in Vero E6 and Calu3 cells. In addition, while we find a slowing of infection in human airway epithelium cultures, we did not observe the near complete inhibition of SARS-CoV-2 replication as seen by Carte-Timofte et al. These findings indicate that inhibition may be cell, virus strain, or model specific, and this should be taken into consideration during screening. Together, our results suggest that atovaquone or other pyrimidine biosynthesis inhibitors could potentially be developed into prophylactic drugs with the potential to slow viral replication, which may limit disease and transmission [53,54].

Our data also show that berberine chloride (BBC) can reduce SARS-CoV-2 replication, similar to what has been found by others [55,56]. BBC was able to block SARS-CoV-2 infectious virus production when added 6 h after the infection was started. This suggests that BBC can act late in the viral life cycle, and could possibly be used to inhibit viral infections after exposure. In addition to replication, we observed that treating SARS-CoV-2 virus directly with BBC was able to significantly reduce infectivity, providing a novel second mode of action for BBC as an antiviral. Importantly, BBC was shown to target a late step in alphavirus replication by inhibiting nucleocapsid formation, as well as reducing virus infectivity by direct effects on viral particles, yet did not inhibit replication of vesicular stomatitis virus (VSV), a negative-strand RNA virus [28]. This observation may suggest that BBC specifically acts on shared mechanisms of the positive-strand RNA virus life cycle, or simply does not work on VSV. It will be interesting if BBC functions through a similar mechanism for SARS-CoV-2 and other positive-strand RNA viruses. Future work will be critical to understand how BBC impacts these distinct viral families. Regardless, the fact that BBC is able to inhibit multiple positive-strand RNA viruses suggests that BBC may be a broad-spectrum antiviral against positive-strand RNA viruses, yet clinical data are needed.

This study identifies the anti-SARS-CoV-2 activities of atovaquone and BBC in vitro. In addition, and equally important, we showed that multiple compounds do not inhibit SARS-CoV-2 in vitro. Together, these points give us insight into some of the host pathways SARS-CoV-2 uses for replication and highlights pathways that we can target for antiviral development. Therefore, it is essential to conduct compound screens for antiviral activity against multiple emerging RNA viruses before the next pandemic.

## Figures and Tables

**Table 1:** Compounds used in this study.

Compound	Source—Catalog Number	Concentrations Tested	Reference
Atorvastatin	TCI America—A24761G	0.625 μM–40 μM	[11,12]
Atovaquone	Tocris Bioscience—6358	0.039 μM–80 μM	[13]
Azithromycin	TCI America—A20761G	0.625 μM–40 μM	[14]
Berberine chloride	Sigma-Aldrich—B3251	10 μM–500 μM	[15]
Brequinar	Sigma-Aldrich—SML0113	0.039 μM–80 μM	[16,17]
Hydroxychloroquine	Santa Cruz—SC-215157	0.625 μM–40 μM	[18]
Imipramine	TCI America—I09711G	0.625 μM–40 μM	[19]
Lovastatin	TCI America—L02145G	0.625 μM–40 μM	[11]
Proguanil hydrochloride	ACROS—461430250	0.039 μM–80 μM	[20]
Ribavirin	Sigma-Aldrich—R9644	6.25 μM–400 μM	[21]
Sofosbuvir	Santa Cruz SC—482362	0.625 μM–40 μM	[22,23]
Verapamil	ACROS Organics—329330010	0.625 μM–40 μM	[24]
Zinc chloride	Fisher—Z33-100	1.56 μM–100 μM	[25]

## Data Availability

Not applicable.

## References

[1] Dittmar M., Lee J.S., Whig K., Segrist E., Li M., Kamalia B., Castellana L., Ayyanathan K., Cardenas-Diaz F.L., Morrisey E.E. (2021). Drug repurposing screens reveal cell-type-specific entry pathways and FDA-approved drugs active against SARS-Cov-2. Cell Rep..

[2] Bakowski M.A., Beutler N., Wolff K.C., Kirkpatrick M.G., Chen E., Nguyen T.-T.H., Riva L., Shaabani N., Parren M., Ricketts J. (2021). Drug repurposing screens identify chemical entities for the development of COVID-19 interventions. Nat. Commun..

[3] Puhl A.C., Fritch E.J., Lane T.R., Tse L.V., Yount B.L., Sacramento C.Q., Fintelman-Rodrigues N., Tavella T.A., Costa F.T.M., Weston S. (2021). Repurposing the ebola and marburg virus inhibitors tilorone, quinacrine, and pyronaridine: In vitro activity against SARS-CoV-2 and potential mechanisms. ACS Omega.

[4] Sheahan T.P., Sims A.C., Graham R.L., Menachery V.D., Gralinski L.E., Case J.B., Leist S.R., Pyrc K., Feng J.Y., Trantcheva I. (2017). Broad-spectrum antiviral GS-5734 inhibits both epidemic and zoonotic coronaviruses. Sci. Transl. Med..

[5] Gordon D.E., Jang G.M., Bouhaddou M., Xu J., Obernier K., White K.M., O’Meara M.J., Rezelj V.V., Guo J.Z., Swaney D.L. (2020). A SARS-CoV-2 protein interaction map reveals targets for drug repurposing. Nature.

[6] Pruijssers A.J., George A.S., Schäfer A., Leist S.R., Gralinksi L.E., Dinnon K.H., Yount B.L., Agostini M.L., Stevens L.J., Chappell J.D. (2020). Remdesivir Inhibits SARS-CoV-2 in human lung cells and chimeric SARS-CoV expressing the SARS-CoV-2 RNA polymerase in mice. Cell Rep..

[7] De Vries M., Mohamed A.S., Prescott R.A., Valero-Jimenez A.M., Desvignes L., O’Connor R., Steppan C., Devlin J.C., Ivanova E., Herrera A. (2021). A comparative analysis of SARS-CoV-2 antivirals characterizes 3CLpro inhibitor PF-00835231 as a potential new treatment for COVID-19. J. Virol..

[8] Harcourt J., Tamin A., Lu X., Kamili S., Sakthivel S.K., Murray J., Queen K., Tao Y., Paden C.R., Zhang J. (2020). Severe acute respiratory syndrome coronavirus 2 from patient with coronavirus disease, United States. Emerg. Infect. Dis..

[9] Xie X., Muruato A., Lokugamage K.G., Narayanan K., Zhang X., Zou J., Liu J., Schindewolf C., Bopp N.E., Aguilar P.V. (2020). An Infectious cDNA Clone of SARS-CoV-2. Cell Host Microbe.

[10] Coffey L.L., Vignuzzi M. (2011). Host alternation of chikungunya virus increases fitness while restricting population diversity and adaptability to novel selective pressures. J. Virol..

[11] Españo E., Nam J.-H., Song E.-J., Song D., Lee C.-K., Kim J.-K. (2019). Lipophilic statins inhibit Zika virus production in Vero cells. Sci. Rep..

[12] Wani M.A., Mukherjee S., Mallick S., Akbar I., Basu A. (2020). Atorvastatin ameliorates viral burden and neural stem/progenitor cell (NSPC) death in an experimental model of Japanese encephalitis. J. Biosci..

[13] Kottkamp A., De Jesus E., Grande R., Brown J.A., Jacobs A.R., Lim J.K., Stapleford K.A. (2019). Atovaquone Inhibits Arbovirus Replication through the Depletion of Intracellular Nucleotides. J. Virol..

[14] Retallack H., Di Lullo E., Arias C., Knopp K.A., Laurie M.T., Sandoval-Espinosa C., Mancia Leon W.R., Krencik R., Ullian E.M., Spatazza J. (2016). Zika virus cell tropism in the developing human brain and inhibition by azithromycin. Proc. Natl. Acad. Sci. USA.

[15] Chen J., Huang X., Tao C., Wang L., Chen Z., Li X., Zeng Q., Ma M., Zhang R., Wu Z. (2020). Berberine chloride suppresses non-small cell lung cancer by deregulating Sin3A/TOP2B pathway in vitro and in vivo. Cancer Chemother. Pharmacol..

[16] Qing M., Zou G., Wang Q.-Y., Xu H.Y., Dong H., Yuan Z., Shi P.-Y. (2010). Characterization of dengue virus resistance to brequinar in cell culture. Antimicrob. Agents Chemother..

[17] Li S.-F., Gong M.-J., Sun Y.-F., Shao J.-J., Zhang Y.-G., Chang H.-Y. (2019). Antiviral activity of brequinar against foot-and-mouth disease virus infection in vitro and in vivo. Biomed. Pharmacother..

[18] Cao B., Parnell L.A., Diamond M.S., Mysorekar I.U. (2017). Inhibition of autophagy limits vertical transmission of Zika virus in pregnant mice. J. Exp. Med..

[19] Wichit S., Hamel R., Bernard E., Talignani L., Diop F., Ferraris P., Liegeois F., Ekchariyawat P., Luplertlop N., Surasombatpattana P. (2017). Imipramine inhibits chikungunya virus replication in human skin fibroblasts through interference with intracellular cholesterol trafficking. Sci. Rep..

[20] Blanshard A., Hine P. (2021). Atovaquone-proguanil for treating uncomplicated Plasmodium falciparum malaria. Cochrane Database Syst. Rev..

[21] Beaucourt S., Vignuzzi M. (2014). Ribavirin: A drug active against many viruses with multiple effects on virus replication and propagation. Molecular basis of ribavirin resistance. Curr. Opin. Virol..

[22] Ferreira A.C., Zaverucha-Do-Valle C., Reis P.A., Barbosa-Lima G., Vieira Y.R., Mattos M., Silva P.D.P., Sacramento C., de Castro Faria Neto H.C., Campanati L. (2017). Sofosbuvir protects Zika virus-infected mice from mortality, preventing short- and long-term sequelae. Sci. Rep..

[23] De Freitas C.S., Higa L.M., Sacramento C.Q., Ferreira A.C., Reis P.A., Delvecchio R., Monteiro F.L., Barbosa-Lima G., Westgarth H.J., Vieira Y.R. (2019). Yellow fever virus is susceptible to sofosbuvir both in vitro and in vivo. PLoS Negl. Trop. Dis..

[24] Nugent K.M., Shanley J.D. (1984). Verapamil inhibits influenza A virus replication. Arch. Virol..

[25] Choi E.-K., Lee H.-H., Kang M.-S., Kim B.-G., Lim H.-S., Kim S.-M., Kang I.-C. (2010). Potentiation of bacterial killing activity of zinc chloride by pyrrolidine dithiocarbamate. J. Microbiol..

[26] Neil J.A., Matsuzawa-Ishimoto Y., Kernbauer-Hölzl E., Schuster S.L., Sota S., Venzon M., Dallari S., Neto A.G., Hine A., Hudesman D. (2019). IFN-I and IL-22 mediate protective effects of intestinal viral infection. Nat. Microbiol..

[27] Matsuzawa-Ishimoto Y., Hine A., Shono Y., Rudensky E., Lazrak A., Yeung F., Neil J.A., Yao X., Chen Y.-H., Heaney T. (2020). An intestinal organoid–based platform that recreates susceptibility to T-cell–mediated tissue injury. Blood.

[28] Wan J.J., Brown R.S., Kielian M. (2020). Berberine chloride is an alphavirus inhibitor that targets nucleocapsid assembly. mBio.

[29] Saib A., Amara W., Wang P., Cattan S., Dellal A., Regaieg K., Nahon S., Nallet O., Nguyen L.S. (2021). Lack of efficacy of hydroxychloroquine and azithromycin in patients hospitalized for COVID-19 pneumonia: A retrospective study. PLoS ONE.

[30] Ulrich R.J., Troxel A.B., Carmody E., Eapen J., Bäcker M., Dehovitz J.A., Prasad P.J., Li Y., Delgado C., Jrada M. (2020). Treating Covid-19 With Hydroxychloroquine (TEACH): A multicenter, double-blind, randomized controlled trial in hospitalized patients. Open Forum Infect. Dis..

[31] Kovacs J. (1992). Efficacy of atovaquone in treatment of toxoplasmosis in patients with AIDS. The NIAID-clinical center intramural AIDS program. Lancet.

[32] Krause P.J., Lepore T., Sikand V.K., Gadbaw J., Burke G., Telford S.R., Brassard P., Pearl D., Azlanzadeh J., Christianson D. (2000). Atovaquone and azithromycin for the treatment of babesiosis. N. Engl. J. Med..

[33] Srivastava I.K., Vaidya A.B. (1999). A Mechanism for the synergistic antimalarial action of atovaquone and proguanil. Antimicrob. Agents Chemother..

[34] Nixon G.L., Pidathala C., Shone A.E., Antoine T., Fisher N., O’Neill P.M., Ward S.A., Biagini G.A. (2013). Targeting the mitochondrial electron transport chain of Plasmodium falciparum: New strategies towards the development of improved antimalarials for the elimination era. Future Med. Chem..

[35] Knecht W., Henseling J., Löffler M. (2000). Kinetics of inhibition of human and rat dihydroorotate dehydrogenase by atovaquone, lawsone derivatives, brequinar sodium and polyporic acid. Chem. Interact..

[36] Ashton T.M., Fokas E., Kunz-Schughart L.A., Folkes L.K., Anbalagan S., Huether M., Kelly C.J., Pirovano G., Buffa F.M., Hammond E.M. (2016). The anti-malarial atovaquone increases radiosensitivity by alleviating tumour hypoxia. Nat. Commun..

[37] Messina E., Gazzaniga P., Micheli V., Guaglianone M.R., Barbato S., Morrone S., Frati L., Aglianò A.M., Giacomello A. (2004). Guanine nucleotide depletion triggers cell cycle arrest and apoptosis in human neuroblastoma cell lines. Int. J. Cancer.

[38] Cohen M.B., Sadee W. (1983). Contributions of the depletions of guanine and adenine nucleotides to the toxicity of purine starvation in the mouse T lymphoma cell line. Cancer Res..

[39] Zang R., Gomez Castro M.F., McCune B.T., Zeng Q., Rothlauf P.W., Sonnek N.M., Liu Z., Brulois K.F., Wang X., Greenberg H.B. (2020). TMPRSS2 and TMPRSS4 promote SARS-CoV-2 infection of human small intestinal enterocytes. Sci. Immunol..

[40] Lamers M.M., Beumer J., van der Vaart J., Knoops K., Puschhof J., Breugem T.I., Ravelli R.B.G., van Schayck J.P., Mykytyn A.Z., Duimel H.Q. (2020). SARS-CoV-2 productively infects human gut enterocytes. Science.

[41] Dickson I. (2020). Organoids demonstrate gut infection by SARS-CoV-2. Nat. Rev. Gastroenterol. Hepatol..

[42] Luganini A., Mercorelli B., Messa L., Palù G., Gribaudo G., Loregian A. (2019). The isoquinoline alkaloid berberine inhibits human cytomegalovirus replication by interfering with the viral Immediate Early-2 (IE2) protein transactivating activity. Antivir. Res..

[43] Hayashi K., Minoda K., Nagaoka Y., Hayashi T., Uesato S. (2007). Antiviral activity of berberine and related compounds against human cytomegalovirus. Bioorg. Med. Chem. Lett..

[44] Wojtyczka R.D., Dziedzic A., Kępa M., Kubina R., Kabała-Dzik A., Mularz T., Idzik D. (2014). berberine enhances the antibacterial activity of selected antibiotics against coagulase-negative staphylococcus strains in vitro. Molecules.

[45] Dziedzic A., Wojtyczka R.D., Kubina R. (2015). Inhibition of oral streptococci growth induced by the complementary action of berberine chloride and antibacterial compounds. Molecules.

[46] Varghese F.S., Thaa B., Amrun S.N., Simarmata D., Rausalu K., Nyman T., Merits A., McInerney G.M., Ng L.F.P., Ahola T. (2016). The antiviral alkaloid berberine reduces chikungunya virus-induced mitogen-activated protein kinase signaling. J. Virol..

[47] Pickard A., Calverley B.C., Chang J., Garva R., Gago S., Lu Y., Kadler K.E. (2021). Discovery of re-purposed drugs that slow SARS-CoV-2 replication in human cells. PLoS Pathog..

[48] Carter-Timofte M.E., Arulanandam R., Kurmasheva N., Fu K., Laroche G., Taha Z., van der Horst D., Cassin L., van der Sluis R.M., Palermo E. (2021). Antiviral potential of the antimicrobial drug atovaquone against SARS-CoV-2 and emerging variants of concern. ACS Infect. Dis..

[49] Calistri A., Luganini A., Mognetti B., Elder E., Sibille G., Conciatori V., Del Vecchio C., Sainas S., Boschi D., Montserrat N. (2021). The New Generation hDHODH Inhibitor MEDS433 Hinders the in vitro replication of SARS-CoV-2 and other human coronaviruses. Microorganisms.

[50] Ko M., Chang S., Byun S., Ianevski A., Choi I., D’Orengiani A.-L.P.H.D., Ravlo E., Wang W., Bjørås M., Kainov D. (2021). Screening of FDA-Approved Drugs Using a MERS-CoV Clinical isolate from south korea identifies potential therapeutic options for COVID-19. Viruses.

[51] Luban J., Sattler R.A., Mühlberger E., Graci J.D., Cao L., Weetall M., Trotta C., Colacino J.M., Bavari S., Strambio-De-Castillia C. (2021). The DHODH inhibitor PTC299 arrests SARS-CoV-2 replication and suppresses induction of inflammatory cytokines. Virus Res..

[52] Bonavia A., Franti M., Keaney E.P., Kuhen K., Seepersaud M., Radetich B., Shao J., Honda A., Dewhurst J., Balabanis K. (2011). Identification of broad-spectrum antiviral compounds and assessment of the druggability of their target for efficacy against respiratory syncytial virus (RSV). Proc. Natl. Acad. Sci. USA.

[53] Yang C.-W., Peng T.-T., Hsu H.-Y., Lee Y.-Z., Wu S.-H., Lin W.-H., Ke Y.-Y., Hsu T.-A., Yeh T.-K., Huang W.-Z. (2020). Repurposing old drugs as antiviral agents for coronaviruses. Biomed. J..

[54] Coelho A.R., Oliveira P.J. (2020). Dihydroorotate dehydrogenase inhibitors in SARS-CoV-2 infection. Eur. J. Clin. Investig..

[55] Wang Z., Li K., Maskey A.R., Huang W., Toutov A.A., Yang N., Srivastava K., Geliebter J., Tiwari R., Miao M. (2021). A small molecule compound berberine as an orally active therapeutic candidate against COVID-19 and SARS: A computational and mechanistic study. FASEB J..

[56] Varghese F., van Woudenbergh E., Overheul G., Eleveld M., Kurver L., van Heerbeek N., van Laarhoven A., Miesen P., Hartog G.D., de Jonge M. (2021). Berberine and Obatoclax Inhibit SARS-Cov-2 Replication in Primary Human Nasal Epithelial Cells In Vitro. Viruses.

